# Clinical characteristics of gut microbiota translocation after acute myocardial infarction and its impact on prognosis

**DOI:** 10.3389/fcvm.2026.1890161

**Published:** 2026-09-10

**Authors:** JunXiang Liu, DongLin Song, Yi Yuan, HaiYing Sun, GuoHong Yang

**Affiliations:** 1 Characteristic Medical Center, Characteristic Medical Center of Chinese People’s Armed Police Force, Tianjin, China; 2 Cardiology Department, Tianjin Provincial Corps Hospital of Chinese People’s Armed Police Forces, Tianjin, China

**Keywords:** acute, gut microbiota, infarction, myocardial, translocation

## Abstract

**Objective:**

To systematically analyze the clinical characteristics of gut microbiota translocation in patients after acute myocardial infarction (AMI), explore the relationships among intestinal barrier dysfunction, gut microbiota changes, inflammatory response, and cardiac function impairment, and further evaluate its association with short-term prognosis, so as to provide new clinical evidence for risk stratification and comprehensive intervention in AMI patients.

**Methods:**

This study adopted a single-center retrospective cohort design. A total of 148 patients with AMI who were hospitalized in the Department of Cardiology of our hospital and received standardized treatment from April 2023 to June 2025 were selected as the study subjects (AMI group), and 110 healthy volunteers who underwent physical examination during the same period were included as the control group. Venous blood and fecal samples had been collected within 24 h after admission or on the day of physical examination as part of the routine clinical laboratory or health examination workflow, and the corresponding laboratory results were retrospectively extracted for this study. Serum D-lactic acid (D-LA), diamine oxidase (DAO), and lipopolysaccharide (LPS) levels were detected by enzyme-linked immunosorbent assay to evaluate intestinal barrier function and microbiota translocation; the quantities of five representative gut microbiota, including Bifidobacterium, Lactobacillus, Bacteroides, Enterobacter, and Enterococcus, were detected by gut microbiota culture. According to serum LPS levels and intestinal barrier indicators, the presence of gut microbiota translocation was comprehensively determined, and patients were divided into a translocation group (*n* = 52) and a non-translocation group (*n* = 96). General clinical data and relevant laboratory indicators were collected, including C-reactive protein (CRP), white blood cell count (WBC), N-terminal pro-brain natriuretic peptide (NT-proBNP), and left ventricular ejection fraction (LVEF). All patients were followed for 90 days after discharge, and newly occurring major adverse cardiovascular events (MACE) during the follow-up period, including heart failure, malignant arrhythmia, recurrent myocardial infarction, and cardiac death, were recorded. Pearson correlation analysis was used to explore the relationships between gut-related indicators and inflammatory indicators as well as cardiac function indicators, and multivariable logistic regression analysis was used to explore factors associated with short-term poor prognosis in patients with AMI.

**Results:**

Compared with the control group, serum D-LA, DAO, and LPS levels were significantly increased in the AMI group (*P* < 0.05). Meanwhile, the gut microbiota structure of AMI patients was markedly disturbed, manifested by a significant decrease in putatively beneficial or commensal bacteria, including Bifidobacterium, Lactobacillus, and Bacteroides, and a significant increase in opportunistic/pathobiont bacteria such as Enterobacter and Enterococcus (P<0.05). Further subgroup analysis showed that, compared with the non-translocation group, the translocation group had more obvious gut microbiota disturbance, characterized by decreased levels of Bifidobacterium, Lactobacillus, and Bacteroides and increased levels of Enterobacter and Enterococcus (*P* < 0.05). Because D-LA, DAO, and LPS were used to define translocation status, their subgroup differences were not interpreted as independent findings. In addition, CRP, WBC, and NT-proBNP levels were significantly higher in the translocation group than in the non-translocation group, whereas LVEF was significantly lower in the translocation group (*P* < 0.05). Follow-up results showed that the incidence of MACE within 90 d was significantly higher in the translocation group than in the non-translocation group, with the most significant increases observed in acute heart failure and malignant arrhythmia (*P* < 0.05). Correlation analysis showed that D-LA, DAO, LPS, Enterobacter, and Enterococcus were significantly positively correlated with CRP, WBC, and NT-proBNP levels and significantly negatively correlated with LVEF (*P* < 0.05); Bifidobacterium, Lactobacillus, and Bacteroides were negatively correlated with CRP, WBC, and NT-proBNP and positively correlated with LVEF (P < 0.05). These correlation results remained statistically significant after Benjamini-Hochberg FDR correction. Exploratory multivariable logistic regression analysis suggested that elevated levels of LPS, DAO, and NT-proBNP, decreased LVEF, and increased abundances of Enterobacter and Enterococcus were associated with poor short-term prognosis in patients with AMI (*P* < 0.05). However, because only 32 patients experienced at least one MACE, these regression results should be interpreted cautiously because of the limited events-per-variable ratio.

**Conclusion:**

AMI patients exhibit marked intestinal barrier dysfunction and gut microbiota translocation during the acute phase, characterized by elevated levels of D-LA, DAO, and LPS, with concomitant decreases in putatively beneficial or commensal bacteria, including Bifidobacterium, Lactobacillus, and Bacteroides, and increases in opportunistic/pathobiont bacteria such as Enterobacter and Enterococcus. The degree of gut microbiota translocation is closely associated with enhanced inflammatory response, worsening cardiac function, and the occurrence of short-term adverse cardiovascular events, suggesting that the “ gut-heart axis “ may play an important role in disease progression and prognostic evolution after AMI. Intestinal barrier function and microbiota translocation-related indicators may serve as potential auxiliary biomarkers for prognostic risk assessment in AMI patients and provide new research directions for future gut microbiota intervention strategies.

## 1 Introduction

Acute myocardial infarction (AMI) is one of the most severe clinical types of coronary atherosclerotic heart disease and is characterized by acute onset, rapid disease progression, and high mortality (1). In recent years, with the continuous development of comprehensive treatment strategies such as percutaneous coronary intervention (PCI), intensified antiplatelet therapy, and lipid-lowering therapy, the short-term survival rate of AMI patients has improved significantly. However, some patients still develop major adverse cardiovascular events (MACE) after AMI, such as heart failure, malignant arrhythmia, recurrent myocardial infarction, and even cardiac death, which seriously affects their long-term prognosis (2). Therefore, further exploration of the mechanisms related to disease progression and poor prognosis after AMI is of great significance for improving clinical outcomes. Previous studies (3, 4) have suggested that the pathophysiological process after AMI is mainly associated with myocardial ischemic necrosis, activation of inflammatory responses, neuroendocrine dysregulation, and ventricular remodeling. In recent years, with the proposal of the “ gut-heart axis “ theory (5), the role of gut microbiota in cardiovascular diseases has gradually attracted attention. As an important microecosystem of the human body, gut microbiota plays an important role in maintaining host metabolic homeostasis, immune regulation, and intestinal barrier function. Under normal circumstances, beneficial bacteria and opportunistic pathogens maintain a dynamic balance and jointly preserve the stability of the intestinal internal environment. When the body is in an acute stress state, the balance of the intestinal microecosystem can easily be disrupted, resulting in microbiota structural disorder and intestinal barrier dysfunction (6, 7).

After AMI, due to decreased cardiac output, excessive activation of the sympathetic nervous system, and intestinal hypoperfusion, intestinal mucosal ischemia, hypoxia, and increased permeability are relatively common, which may further cause gut microbiota and their metabolites to break through the intestinal mucosal barrier and enter the bloodstream, namely “gut microbiota translocation.” Studies (8, 9) have shown that gut microbiota translocation can lead to the entry of bacterial endotoxins such as lipopolysaccharide (LPS) into the blood, activate inflammatory signaling pathways, and induce the release of large amounts of inflammatory factors, thereby further aggravating myocardial injury and cardiac function deterioration. Meanwhile, intestinal barrier impairment may also form a vicious cycle of “ inflammatory response—intestinal injury—cardiac function deterioration ” (10), participating in the occurrence and development of poor prognosis after AMI. At present, studies on gut microbiota changes in AMI patients are gradually increasing, but most mainly focus on changes in microbiota structure and metabolites, while clinical studies on the relationship between intestinal barrier dysfunction, microbiota translocation, and prognosis in AMI patients remain relatively limited. D-LA, diamine oxidase (DAO), and lipopolysaccharide (LPS) are currently important indicators for evaluating intestinal barrier function and microbiota translocation. Among them, elevated D-LA indicates increased intestinal mucosal permeability; DAO can reflect intestinal mucosal epithelial cell injury; and LPS is considered important indirect evidence of bacterial translocation. In addition, genus-level changes such as decreases in Bifidobacterium, Lactobacillus, and Bacteroides, as well as increases in Enterobacter and Enterococcus, are considered representative manifestations of intestinal microecological imbalance. Based on this background, this study detected intestinal barrier function-related indicators and representative gut microbiota changes in AMI patients, analyzed the clinical characteristics of gut microbiota translocation after AMI, and further explored its relationships with inflammatory response, cardiac function impairment, and short-term prognosis, aiming to provide a new theoretical basis for risk assessment and gut microbiota intervention in AMI patients.

## 2 Materials and methods

### 2.1 Study design and study subjects

This study was a single-center retrospective cohort study conducted based on real-world clinical and laboratory data that had been generated during routine diagnosis, treatment, and health examination workflows. A total of 148 AMI patients who were hospitalized in the Department of Cardiology of our hospital and received standardized treatment from April 2023 to June 2025 were enrolled as the AMI group, while 110 healthy volunteers who underwent physical examination at the physical examination center of our hospital during the same period were selected as the control group. In the AMI group, there were 94 males and 54 females, aged 41–82 years, with a mean age of (63.14 ± 10.27) years; body mass index (BMI) was (24.36 ± 2.71) kg/m^2^; 61 patients (41.22%) had a smoking history, and 46 patients (31.08%) had a drinking history. In the healthy control group, there were 68 males and 42 females, aged 39–79 years, with a mean age of (61.72 ± 9.85) years; BMI was (23.98 ± 2.54) kg/m^2^; 36 participants (32.73%) had a smoking history, and 29 participants (26.36%) had a drinking history. There were no statistically significant differences between the two groups in general demographic characteristics such as sex composition, age, BMI, smoking history, and drinking history (*P* > 0.05), indicating good comparability between the two groups.

Sample size consideration: Because this was a single-center retrospective cohort study based on real-world clinical data, no formal *a priori* sample size calculation was performed before patient enrollment. The sample size was determined by the number of eligible subjects with complete clinical, laboratory, gut microbiota, and 90-d follow-up data during the predefined study period from April 2023 to June 2025. A total of 148 AMI patients and 110 healthy controls met the inclusion and exclusion criteria and were included in the final analysis. The subgroup sample sizes of the translocation group (*n* = 52) and non-translocation group (*n* = 96) were determined according to the predefined criteria for gut microbiota translocation rather than by random allocation or prospective sample size planning. Therefore, subgroup and prognostic analyses, particularly the exploratory Logistic regression analysis based on 32 MACE events, should be interpreted cautiously.

This study strictly adhered to the principles of the Declaration of Helsinki and the ethical standards for biomedical research in China. This study was conducted in accordance with the Declaration of Helsinki and was approved by the Ethics Committee of the Characteristic Medical Center of Chinese People's Armed Police Forces (approval No. Clinical Research 2023-0016.1; project No. Clinical Research 2023-0016). Because this retrospective study used de-identified clinical and laboratory data generated during routine medical care and health examinations, without additional intervention or study-specific sample collection, the requirement for written informed consent was waived by the Ethics Committee.

**Figure 1:**
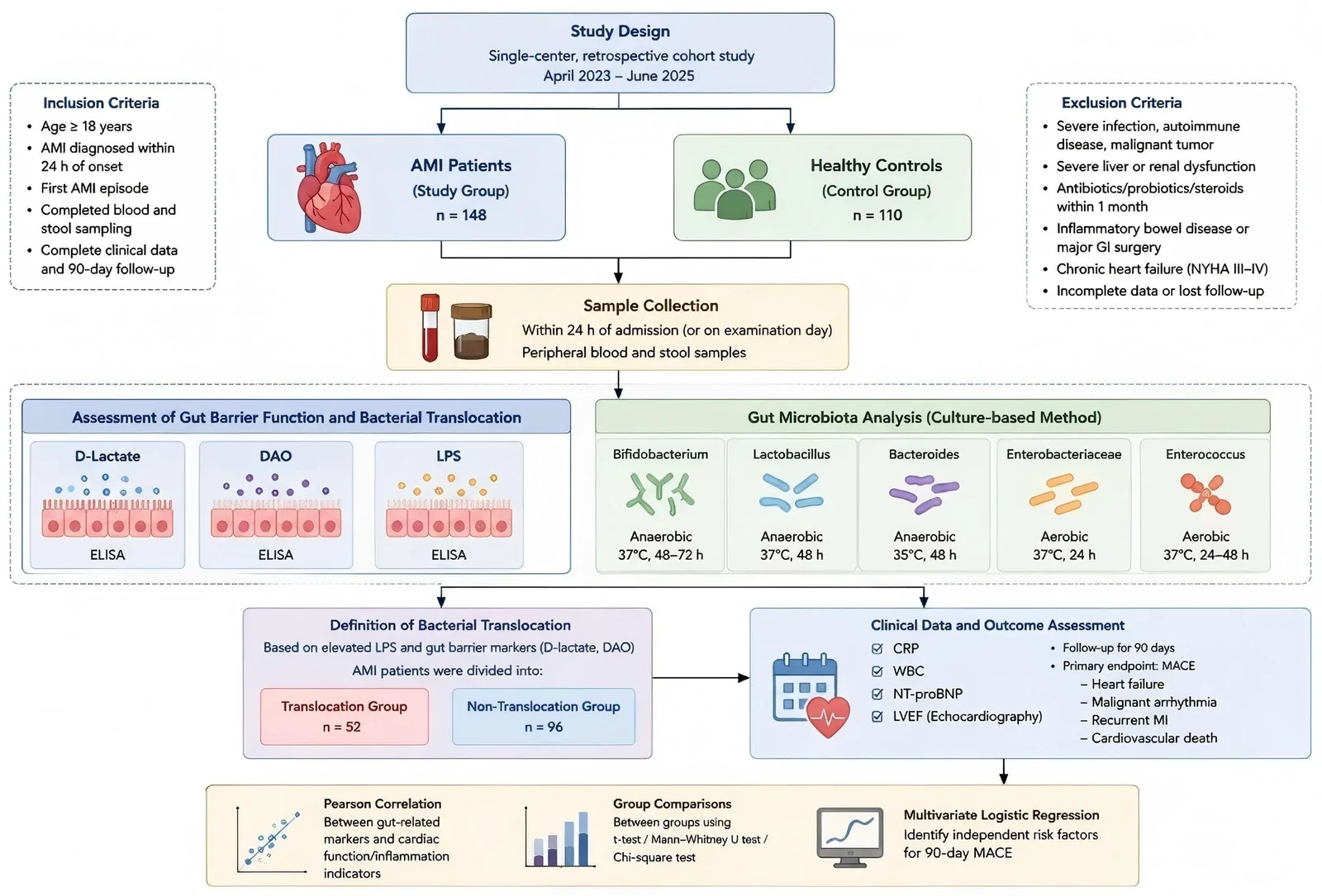
Flowchart of study design, participant enrollment, gut microbiota assessment, and outcome evaluation in patients with acute myocardial infarction.

All study data, including laboratory results of intestinal barrier indicators and gut microbiota culture, were obtained from the hospital electronic medical record system, laboratory information system, and follow-up database. During the study process, patients ’ names, hospitalization numbers, and identification information were de-identified, and only authorized researchers had access to relevant data to ensure patient privacy and data security. The overview flowchart of this study is shown in Figure 1.

### 2.2 Inclusion and exclusion criteria

#### 2.2.1 Inclusion criteria for the AMI group

(1) Patients met the relevant clinical diagnostic criteria for AMI (11), and the diagnosis was confirmed based on typical clinical symptoms, dynamic electrocardiographic changes, and elevated myocardial injury biomarkers; (2) Age ≥ 18 years, regardless of sex; (3) First occurrence of AMI and admission within 24 h after onset; (4) Availability of routinely performed gut microbiota and intestinal barrier function-related test results during hospitalization; (5) Complete clinical data and availability of 90-d follow-up results.

#### 2.2.2 Inclusion criteria for the control group

(1) Healthy individuals who underwent physical examination at the physical examination center of our hospital during the same period; (2) No definite history of coronary heart disease, heart failure, or other severe cardiovascular diseases; (3) No severe hepatic, renal, gastrointestinal, or infectious diseases; (4) No use of antibiotics, probiotics, or intestinal microecological preparations within the previous month; (5) Generally normal physical examination and routine laboratory test results, with available gut microbiota and intestinal barrier-related laboratory records from the health examination.

#### 2.2.3 Exclusion criteria

(1) Patients previously diagnosed with chronic heart failure or severe cardiac insufficiency; (2) Patients complicated with severe infectious diseases, autoimmune diseases, malignant tumors, or hematological diseases; (3) Patients complicated with severe hepatic or renal insufficiency; (4) Use of antibiotics, probiotics, glucocorticoids, or intestinal microecological preparations within the previous month; (5) Patients complicated with inflammatory bowel disease, severe digestive system diseases, or a history of gastrointestinal surgery; (6) Long-term alcohol abuse or severe malnutrition; (7) Incomplete clinical data or loss to follow-up.

### 2.3 Research methods

#### 2.3.1 Collection of general clinical data

General clinical data and relevant laboratory and examination results were retrospectively extracted from the hospital electronic medical record system and laboratory information system. All data were independently entered and verified by two uniformly trained researchers to ensure the accuracy and completeness of the data. The collected information mainly included: (1) general demographic data, including age, sex, BMI, smoking history, and drinking history; (2) underlying diseases and past medical history, including hypertension, diabetes mellitus, hyperlipidemia, coronary heart disease, stroke, and chronic kidney disease, as well as long-term medication use and family history of cardiovascular diseases; (3) AMI-related clinical data, including onset time, infarction location, STEMI/NSTEMI classification, Killip classification (12), whether emergency PCI was performed, and culprit vessel conditions; (4) medication treatment during hospitalization, including routine treatment regimens such as antiplatelet drugs, *β*-blockers, ACEI/ARB, and statins.

#### 2.3.2 Fecal sample collection and gut microbiota detection

(1) Fecal sample collection: In the hospital routine clinical laboratory workflow, fresh naturally excreted fecal samples (approximately 3–5 g) had been collected within 24 h after admission for AMI patients who underwent gut microbiota and intestinal barrier-related testing, and on the day of physical examination for eligible healthy controls who received the corresponding health examination tests. The samples were placed into pre-labeled sterile fecal preservation tubes, avoiding contamination by urine or other impurities. After collection, the samples were immediately sealed and transported to the microbiology laboratory of the Department of Clinical Laboratory within 30 min for routine processing. For the present retrospective study, no additional fecal samples were collected; only existing laboratory records and culture results were extracted and analyzed. (2) Fecal sample processing and gradient dilution: All samples were processed under sterile conditions. One gram of fecal sample was accurately weighed, mixed with 9 mL sterile normal saline or phosphate-buffered saline (PBS), and thoroughly shaken to prepare a fecal suspension. Subsequently, ten-fold serial gradient dilution was performed stepwise to concentrations ranging from 10 to 7 to 10-8. (3) Gut microbiota culture and identification: Five representative gut microbiota, including Bifidobacterium, Lactobacillus, Bacteroides, Enterobacter, and Enterococcus, were selected for detection in this study. In this study, Bifidobacterium and Lactobacillus were regarded as representative putatively beneficial bacteria because of their commonly reported roles in maintaining intestinal microecological stability and barrier function. Bacteroides was analyzed as a representative anaerobic commensal genus rather than being uniformly defined as beneficial at the species or strain level, because different Bacteroides species or strains may have distinct biological effects. Enterobacter and Enterococcus were interpreted as opportunistic/pathobiont bacteria in the context of intestinal dysbiosis. These classifications were used only for descriptive interpretation at the genus level, and the culture-based method used in this study did not allow species- or strain-level functional identification. A total of 100 μL of fecal suspension from different dilution gradients was inoculated onto the surfaces of corresponding selective culture media and evenly spread using sterile spreaders. For anaerobic culture, the inoculated plates for Bifidobacterium, Lactobacillus, and Bacteroides were immediately placed into sealed anaerobic jars. Anaerocult ™ A gas-generating sachets (Merck KGaA, Darmstadt Germany; catalog number: 1. 13829) were used to remove residual oxygen and establish an anaerobic atmosphere within the jars. Anaerotest® indicator strips (Merck KGaA, Darmstadt, Germany; catalog number: 1.32371) were placed in each jar to confirm that anaerobic conditions had been successfully established and maintained throughout the incubation period. The jars were tightly sealed immediately after sample loading and were not opened during incubation to minimize oxygen exposure. Different culture conditions were then established according to the growth characteristics of each microbiota:

① Bifidobacterium: cultured using Bifidobacterium selective medium (Qingdao Hope Bio-Technology Co., Ltd., catalog number: HB8527) under anaerobic conditions at 37 ℃ for 48–72 h;

② Lactobacillus: cultured using MRS medium (Qingdao Hope Bio-Technology Co., Ltd., catalog number: HB0384) under anaerobic conditions at 37 ℃ for 48 h;

③ Bacteroides: cultured using Bacteroides selective medium (Qingdao Hope Bio-Technology Co., Ltd., catalog number: HB7028) under anaerobic conditions at 35 ℃ for 48 h;

④ Enterobacter: cultured using MacConkey agar medium (Qingdao Hope Bio-Technology Co., Ltd., catalog number: HB8458) under aerobic conditions at 37 ℃ for 24 h;

⑤ Enterococcus: cultured using bile esculin agar medium (Sigma-Aldrich, catalog number: CM0888B) under aerobic conditions at 37 ℃ for 24–48 h. After culture, colony counting and bacterial identification were independently performed by two laboratory technicians. Suspicious colonies were further identified by Gram staining and biochemical reactions. Because this retrospective study was based on existing routine clinical laboratory records, PCR-based detection of genes involved in beneficial metabolite production or strain-specific molecular markers was not available. (4) Colony counting and result expression: Plates with colony counts ranging from 30 to 300 were selected for counting. The number of colony-forming units (CFU) per gram of feces was calculated according to the dilution factor, and the final results were expressed as log(CFU/g). All samples were cultured repeatedly, and the average value was taken as the final result.

#### 2.3.3 Detection and determination of intestinal barrier function and gut microbiota translocation indicators

According to the routine admission laboratory protocol, AMI patients had 5 mL of fasting venous blood collected within 24 h after admission and placed into procoagulant tubes. These tests had been completed by the Department of Clinical Laboratory during hospitalization, and the results were retrospectively extracted for analysis. After standing at room temperature for 30 min, the samples were centrifuged at 3,000 rpm for 10 min. The supernatant serum was separated and stored at −80 °C for subsequent testing. Serum D-LA, DAO, and LPS levels were detected by enzyme-linked immunosorbent assay (ELISA). Among them, the D-LA detection kit (catalog number: CB10127-Hu), DAO detection kit (catalog number: CB10538-Hu), and LPS detection kit (catalog number: CB11733-Hu) were all purchased from Shanghai Kehai Biotechnology Co., Ltd. All procedures were performed strictly according to the manufacturers ‘ instructions and completed uniformly by the Department of Clinical Laboratory of our hospital. The normal reference ranges of the above indicators were determined according to the standards of the Department of Clinical Laboratory and kit instructions, with normal reference values defined as D-LA＜ 15 mg/L, DAO＜ 10 U/L, and LPS＜20 pg/mL. Values exceeding the above ranges were defined as abnormally elevated. The presence of gut microbiota translocation was comprehensively determined according to serum LPS levels and intestinal barrier function-related indicators. Patients who simultaneously met the following conditions were defined as having gut microbiota translocation: (1) LPS level higher than the normal reference range; (2) elevated levels of at least one of D-LA or DAO. According to the above criteria, AMI patients were divided into a translocation group (*n* = 52) and a non-translocation group (*n* = 96). Because serum LPS and intestinal barrier indicators were used to define group assignment, subsequent between-group comparisons of D-LA, DAO, and LPS were considered only as verification of the grouping procedure and were not interpreted as independent study findings. The main subgroup analyses therefore focused on gut microbiota indicators, inflammatory/cardiac function indicators, clinical outcomes, and continuous correlation analyses.

#### 2.3.4 Detection of inflammatory and cardiac function indicators

1. Detection of inflammatory indicators: All AMI patients had fasting venous blood samples collected within 24 h after admission, and related inflammatory indicators were uniformly detected by the Department of Clinical Laboratory of our hospital. C-reactive protein (CRP) levels were measured using an automatic biochemical analyzer (AU5800, Beckman Coulter, USA); white blood cell count (WBC) levels were measured using an automatic hematology analyzer (XN-9000, Sysmex Corporation, Japan). All tests were performed strictly in accordance with instrument operating procedures and reagent instructions, and regular internal quality control was conducted to ensure the accuracy and stability of the test results.
2. Detection of cardiac function indicators: ① Detection of N-terminal pro-brain natriuretic peptide (NT-proBNP): All AMI patients had 3–5 mL of fasting venous blood collected within 24 h after admission. Serum was separated after centrifugation, and NT-proBNP levels were measured by chemiluminescence immunoassay. The detection instrument used was a Cobas e601 automatic electrochemiluminescence analyzer (Roche Diagnostics, Germany), and the related reagent kits were provided as matching reagents for the instrument. All tests were completed by full-time personnel from the Department of Clinical Laboratory according to standard operating procedures. ② Measurement of left ventricular ejection fraction (LVEF): All AMI patients underwent routine echocardiographic examination during hospitalization using a color Doppler ultrasound diagnostic system (Vivid E95, GE Healthcare, USA). Image acquisition and measurements were independently completed by two experienced sonographers. Patients were placed in the left lateral decubitus position and examined at rest. The Simpson biplane method was used to measure left ventricular end-diastolic volume and end-systolic volume, and LVEF was subsequently calculated. All indicators were measured three consecutive times, and the average value was taken as the final result.

### 2.4 Follow-up methods

All AMI patients were followed up for 90 days after discharge through a combination of outpatient follow-up and telephone follow-up. The follow-up work was conducted by fixed research personnel, and a unified follow-up registration form was established to ensure the completeness and consistency of follow-up data. The first follow-up was conducted 30 d after discharge, and the final follow-up was completed at 90 d; for patients with significant disease progression or special conditions, the frequency of follow-up was appropriately increased. During the follow-up process, emphasis was placed on recording cardiac function recovery, rehospitalization, and the occurrence of major adverse cardiovascular events (MACE). The primary endpoint events included: (1) acute heart failure; (2) malignant arrhythmia; (3) recurrent myocardial infarction; and (4) cardiac death. All endpoint events were independently evaluated by two senior cardiovascular specialists according to patients ‘ medical records, re-examination results, and follow-up records. During endpoint adjudication, the two specialists were blinded to the patients’ translocation grouping and gut-related laboratory or culture results. If disagreements occurred, a consensus conclusion was reached after discussion. For patients lost to follow-up, additional information was supplemented through repeated telephone contact or review of the hospitalization system to minimize follow-up bias as much as possible.

### 2.5 Statistical methods

Given the retrospective nature of the study, no formal *a priori* power calculation was performed, and the analyses were conducted to evaluate clinical associations within the available dataset. Continuous variables were assessed for distributional characteristics before analysis. Variables approximately conforming to a normal distribution were expressed as mean ± standard deviation and compared using the independent-samples t test, whereas variables with non-normal distributions were expressed as median (interquartile range) and compared using the Mann–Whitney U test. Before final analysis, the distributions of the main continuous variables were reviewed for obvious data-entry errors, duplicated records, implausible values, or extreme outliers. Count data were expressed as number of cases (%), and comparisons between groups were performed using the chi-square test or Fisher 's exact test.

Pearson correlation analysis was used to evaluate the relationships between gut-related indicators and inflammatory/cardiac function indicators. A heatmap was generated to visually summarize the direction and magnitude of the Pearson correlation coefficients. To account for multiple statistical testing, the Benjamini-Hochberg false discovery rate (FDR) correction was applied to the main multiple-comparison analyses and to the 32 Pearson correlation tests in Table 5.

Nominal *P*-values were reported in the main tables, whereas FDR-adjusted *P*-values were reported as q values in Supplementary Table S1. A q value < 0.05 was considered statistically significant after FDR correction. Multivariable Logistic regression analysis was performed as an exploratory analysis to evaluate factors associated with short-term poor prognosis in AMI patients. Because only 32 patients experienced at least one MACE during the 90-d follow-up, the regression results were interpreted cautiously considering the limited events-per-variable ratio. A *P*-value < 0.05 was considered statistically significant for unadjusted analyses.

## 3 Results

Before final statistical analysis, the main continuous variables were reviewed for distributional characteristics, and no obvious data-entry errors or biologically implausible values were identified. To address multiple testing, FDR-adjusted q values were calculated for the main multiple comparisons and correlation analyses.

Specifically, the 32 Pearson correlation tests in Table 5 were adjusted using the Benjamini-Hochberg FDR method. The statistically significant findings reported in the main analyses remained significant after FDR correction. Detailed statistical test results, including test methods, test statistics, nominal *P* -values, and FDR-adjusted q values where applicable, are provided in Supplementary Table S1.

### 3.1 Comparison of intestinal barrier function and gut microbiota between the AMI group and the control group

**Figure 2:**
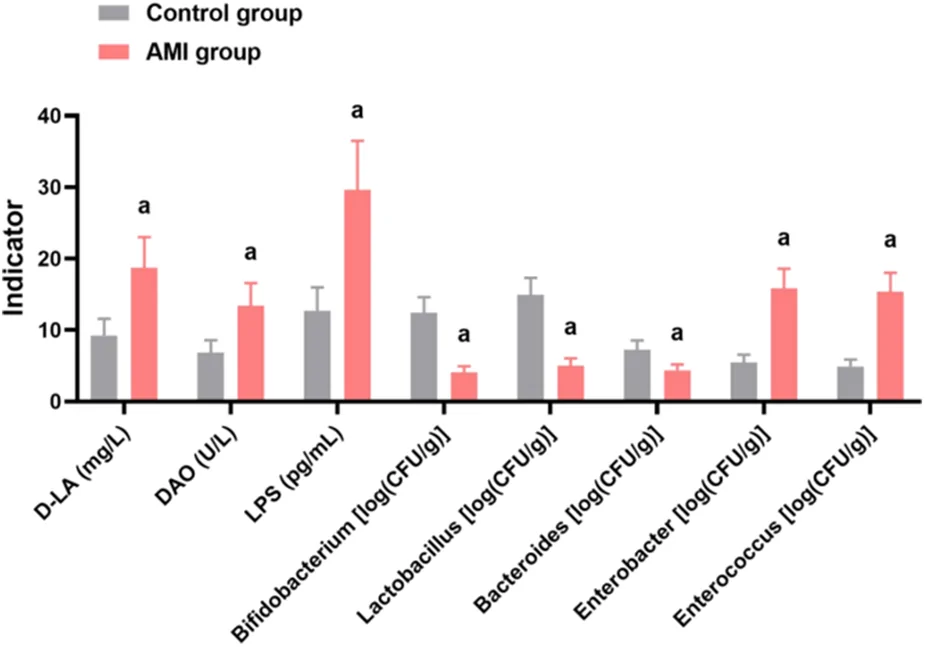
Comparison of intestinal barrier function and Gut Microbiota between the AMI group and the control group data are presented as mean ± standard deviation. This figure was used as a concise group-level summary visualization; detailed group means, standard deviations, test statistics, and *P*-values are shown in Table 1, and additional statistical details are provided in Supplementary Table S1. a indicates *P* < 0.05 for intergroup comparison.

Compared with the control group, serum D-LA, DAO, and LPS levels were significantly elevated in the AMI group (*P* < 0.05). Meanwhile, the gut microbiota structure in the AMI group was markedly disturbed, manifested by significantly decreased numbers of putatively beneficial or commensal bacteria, including Bifidobacterium, Lactobacillus, and Bacteroides, whereas the numbers of opportunistic/pathobiont bacteria such as Enterobacter and Enterococcus were significantly increased (*P* < 0.05) (Table 1 and Figure 2).

**Table 1:** Comparison of intestinal barrier function and Gut Microbiota between the AMI group and the control group.

Indicators	Control group (*n* = 110)	AMI group (*n* = 148)	t	P
D-LA (mg/L)	9.26 ± 2.31	18.74 ± 4.28	21.056	<0.001
DAO (U/L)	6.84 ± 1.75	13.42 ± 3.15	19.753	<0.001
LPS (pg/mL)	12.73 ± 3.26	29.65 ± 6.84	23.989	<0.001
Bifidobacterium [log(CFU/g)]	12.45 ± 2.16	4.08 ± 0.86	42.817	<0.001
Lactobacillus [log(CFU/g)]	14.92 ± 2.38	5.02 ± 1.03	45.245	<0.001
Bacteroides [log(CFU/g)]	7.26 ± 1.28	4.37 ± 0.81	22.148	<0.001
Enterobacter [log(CFU/g)]	5.48 ± 1.07	15.84 ± 2.73	37.692	<0.001
Enterococcus [log(CFU/g)]	4.92 ± 0.95	15.39 ± 2.61	40.127	<0.001

### 3.2 Comparison of general clinical data between the translocation group and the non-translocation group

According to the diagnostic criteria for gut microbiota translocation, 148 AMI patients were divided into a translocation group (*n* = 52) and a non-translocation group (*n* = 96).

The results showed no statistically significant differences between the two groups in sex composition, age, BMI, smoking history, drinking history, or underlying diseases such as hypertension, diabetes mellitus, and hyperlipidemia (*P* > 0.05), indicating good comparability in general clinical baseline characteristics between the two groups.

In addition, there were no statistically significant differences between the two groups in STEMI/NSTEMI classification, Killip classification, emergency PCI implementation, culprit vessels, or major medication regimens during hospitalization (P > 0.05) (Table 2).

**Table 2:** Comparison of general clinical data between the translocation group and the Non-translocation group.

Characteristic	Non-translocation group (*n* = 96)	Translocation group (*n* = 52)	t/x2	P
Sex	-	-	0.121	0.727
Male	60 (62.50%)	34 (65.38%)	-	-
Female	36 (37.50%)	18 (34.62%)	-	-
Age (years)	62.71 ± 9.84	63.93 ± 10.58	0.695	0.487
BMI (kg/m2)	24. 18 ± 2.62	24.69 ± 2.77	1.093	0.272
Smoking history			0.763	0.382
Yes	39 (40.63%)	25 (48.08%)	-	-
No	57 (59.37%)	27 (51.92%)	-	-
Drinking history	-	-	0.467	0.494
Yes	28 (29. 17%)	18 (34.62%)	-	-
No	68 (70.83%)	34 (65.38%)	-	-
Underlying diseases	-	-	-	-
Hypertension	41 (42.71%)	26 (50.00%)	0.723	0.394
Diabetes mellitus	28 (29. 17%)	18 (34.62%)	0.467	0.494
Hyperlipidemia	31 (32.29%)	20 (38.46%)	0.568	0.450
STEMI	-	-	0.470	0.492
Yes	63 (65.63%)	37 (71. 15%)	-	-
No	33 (34.38%)	15 (28.85%)	-	-
Killip classification	-	-	0.724	0.394
Grade I	42 (43.75%)	19 (36.54%)	-	-
Grade II	36 (37.50%)	22 (42.31%)	-	-
Grade III	13 (13.54%)	8 (15.38%)	-	-
Grade IV	5 (5.21%)	3 (5.77%)	-	-
Emergency PCI	-	-	0.261	0.609
Yes	79 (82.29%)	41 (78.85%)	-	-
No	17 (17.71%)	11 (21. 15%)	-	-
Culprit vessel	-	-	0.474	0.491
Right coronary artery	31 (32.29%)	16 (30.77%)	-	-
Left anterior descending artery	46 (47.92%)	28 (53.85%)	-	-
Left circumflex artery	19 (19.79%)	8 (15.38%)	-	-
In-hospital medication	-	-	-	-
*β*-blockers	73 (76.04%)	38 (73.08%)	0.158	0.690
ACEI/ARB	71 (73.96%)	37 (71. 15%)	0.134	0.713
Statins	88 (91.67%)	46 (88.46%)	0.116	0.732

### 3.3 Comparison of gut microbiota between the translocation group and the Non-translocation group

**Figure 3:**
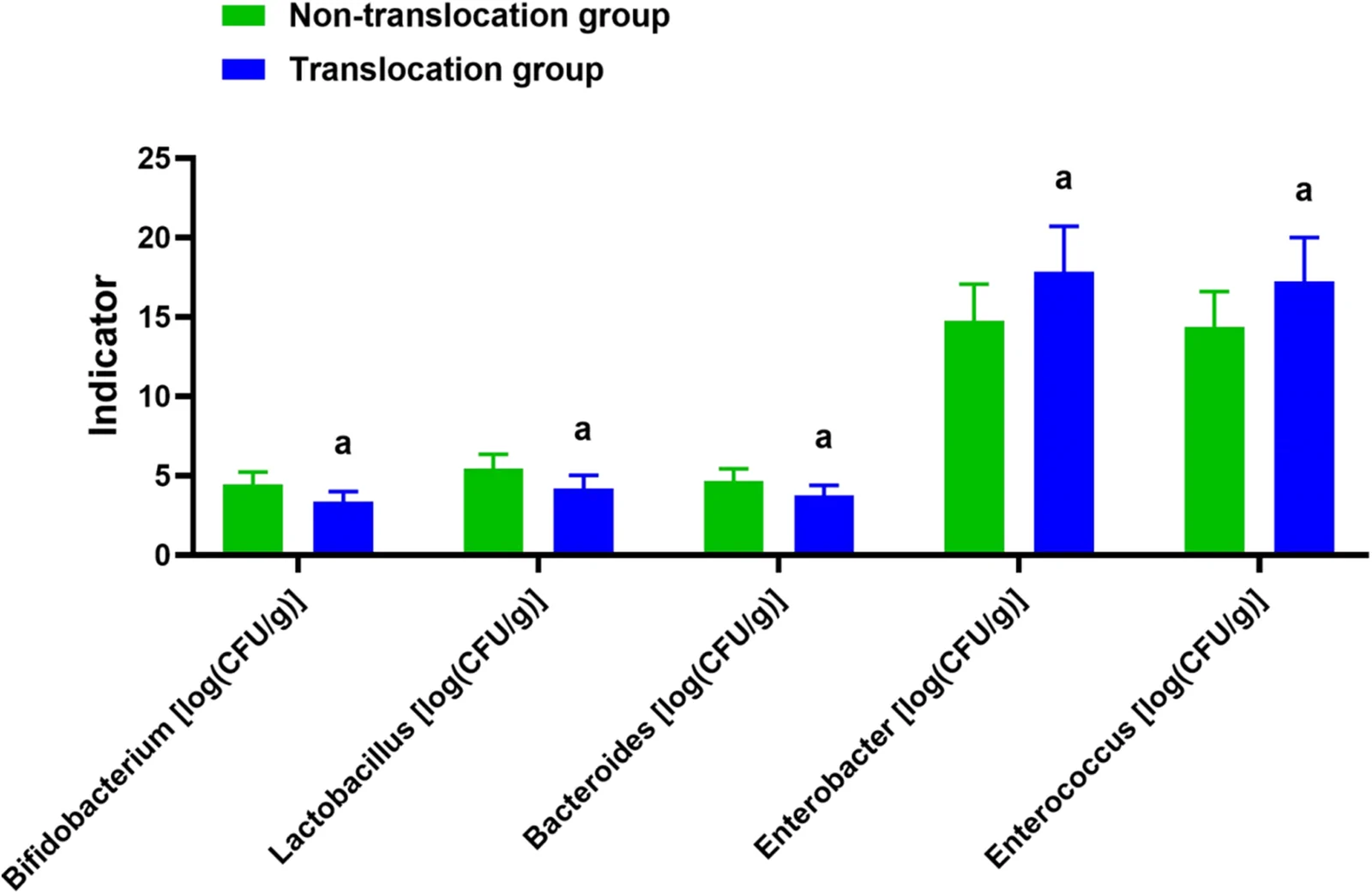
Comparison of gut microbiota between the translocation group and the non-translocation group. Data are presented as mean ± standard deviation. This figure was used as a concise group-level summary visualization; detailed group means, standard deviations, test statistics, and *P*-values are shown in Table 3, and additional statistical details are provided in Supplementary Table S1. a indicates *P* < 0.05 for intergroup comparison.

Because serum LPS and intestinal barrier indicators were used to define translocation status, differences in D-LA, DAO, and LPS between the translocation and non-translocation groups were expected by design and were not treated as independent outcome findings. Therefore, the subgroup comparison in this section focused on gut microbiota indicators. Compared with the non-translocation group, the translocation group showed significantly decreased levels of Bifidobacterium, Lactobacillus, and Bacteroides, whereas the levels of Enterobacter and Enterococcus were significantly increased (*P* < 0.05) (Table 3 and Figure 3).

**Table 3:** Comparison of Gut Microbiota between the translocation group and the Non-translocation group.

Indicators	Non-translocation group (*n* = 96)	Translocation group (*n* = 52)	t	P
Bifidobacterium [log(CFU/g)]	4.46 ± 0.78	3.38 ± 0.64	8.543	＜0.001
Lactobacillus [log(CFU/g)]	5.46 ± 0.91	4.21 ± 0.82	8.253	＜0.001
Bacteroides [log(CFU/g)]	4.69 ± 0.75	3.78 ± 0.63	7.439	＜0.001
Enterobacter [log(CFU/g)]	14.76 ± 2.31	17.84 ± 2.87	7.098	＜0.001
Enterococcus [log(CFU/g)]	14.38 ± 2.24	17.25 ± 2.75	6.858	＜0.001

### 3.4 Comparison of inflammatory indicators, cardiac function indicators, and 90-d cardiovascular outcomes between the translocation group and the Non-translocation group

The comparison of inflammatory indicators showed that CRP and WBC levels in the translocation group were significantly higher than those in the non-translocation group (*P* < 0.05). Comparison of cardiac function indicators showed that NT-proBNP levels in the translocation group were significantly higher, whereas LVEF was significantly lower than those in the non-translocation group (*P* < 0.05). Follow-up results showed that the overall incidence of MACE within 90 d was significantly higher in the translocation group than in the non-translocation group (34.62% vs. 14.58%, *P* < 0.05), among which the incidences of acute heart failure and malignant arrhythmia increased most significantly (Table 4).

**Table 4:** Comparison of inflammatory indicators, cardiac function indicators, and 90-d prognosis between the translocation group and the Non-translocation group.

Indicators	Non-translocation group (*n* = 96)	Translocation group (*n* = 52)	t/x2	P
CRP (mg/L)	18.65 ± 5.72	31.84 ± 8.46	11.259	<0.001
WBC (×10⁹/L)	9. 18 ± 2.11	13.42 ± 3.26	9.578	<0.001
NT-proBNP (ng/L)	1186.37 ± 235.48	1764.82 ± 356.73	11.838	<0.001
LVEF (%)	56.24 ± 7.16	47.82 ± 6.41	7.079	<0.001
Total incidence of MACE	14 (14.58%)	18 (34.62%)	7.986	0.004
Acute heart failure	7 (7.29%)	11 (21. 15%)	6.067	0.013
Malignant arrhythmia	4 (4. 17%)	8 (15.38%)	4.290	0.038
Recurrent myocardial infarction	2 (2.08%)	4 (7.69%)	1.476	0.224
Cardiac death	1 (1.04%)	3 (5.77%)	1.350	0.245

### 3.5 Correlation analysis between gut-related indicators and inflammatory indicators as well as cardiac function indicators

**Figure 4:**
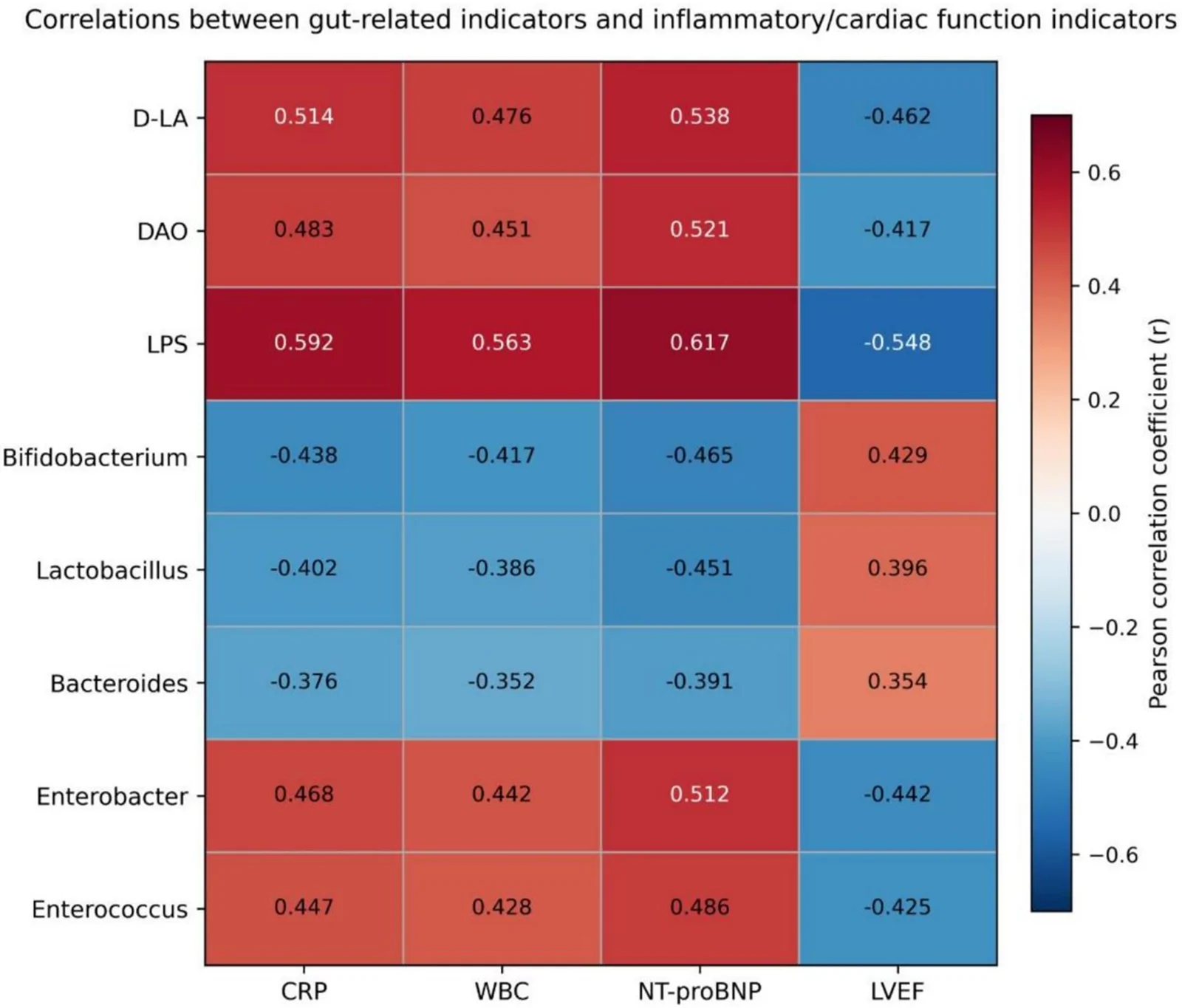
Heatmap of correlations between gut-related indicators and inflammatory/cardiac function indicators in patients with acute myocardial infarction. The heatmap shows Pearson correlation coefficients between gut-related indicators and CRP, WBC, NT-proBNP, and LVEF. The color scale indicates the direction and magnitude of the correlations. CRP, C-reactive protein; WBC, white blood cell count; NT-proBNP, N-terminal pro-brain natriuretic peptide; LVEF, left ventricular ejection fraction.

**Figure 5:**
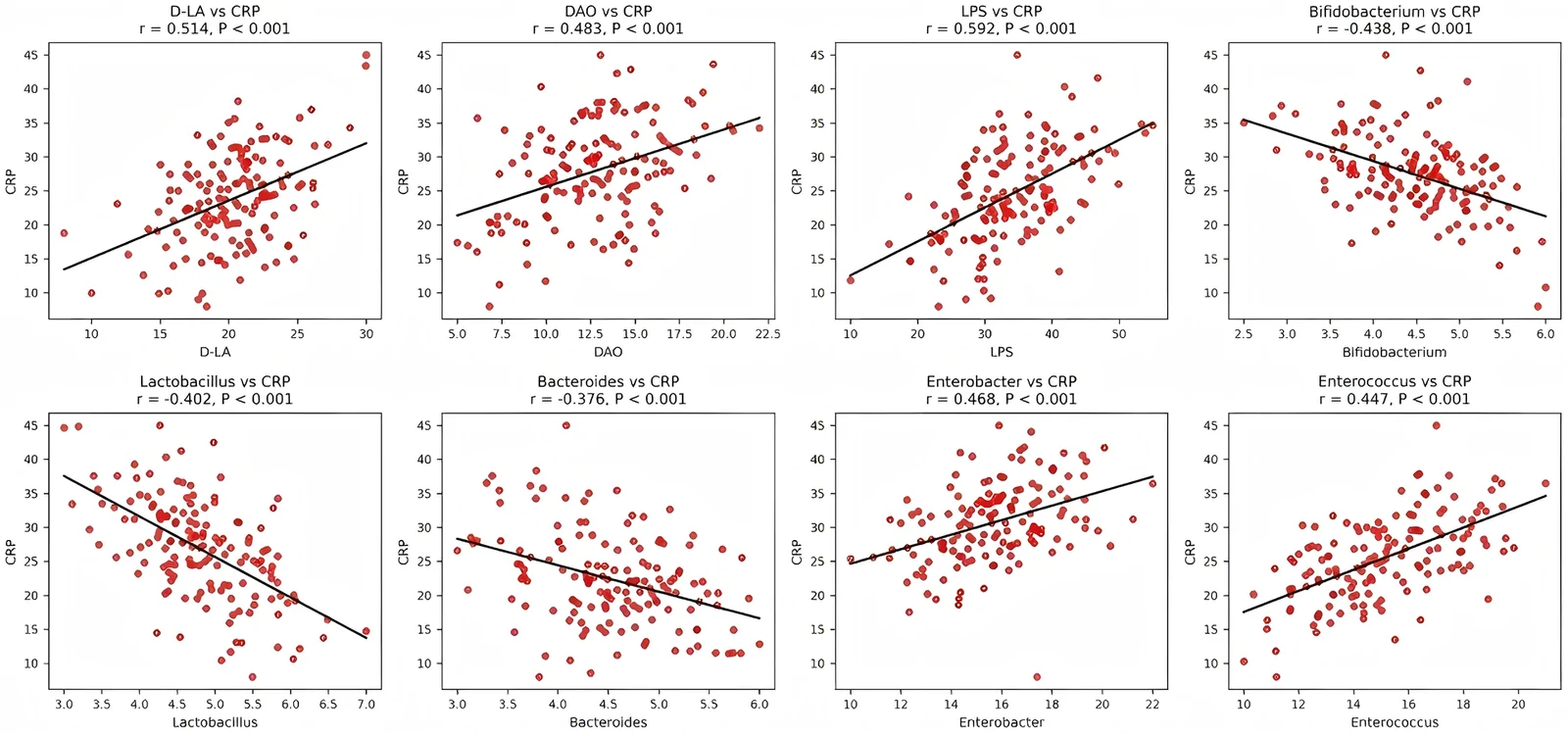
Correlation between gut-related indicators and CRP levels in patients with acute myocardial infarction.

**Figure 6:**
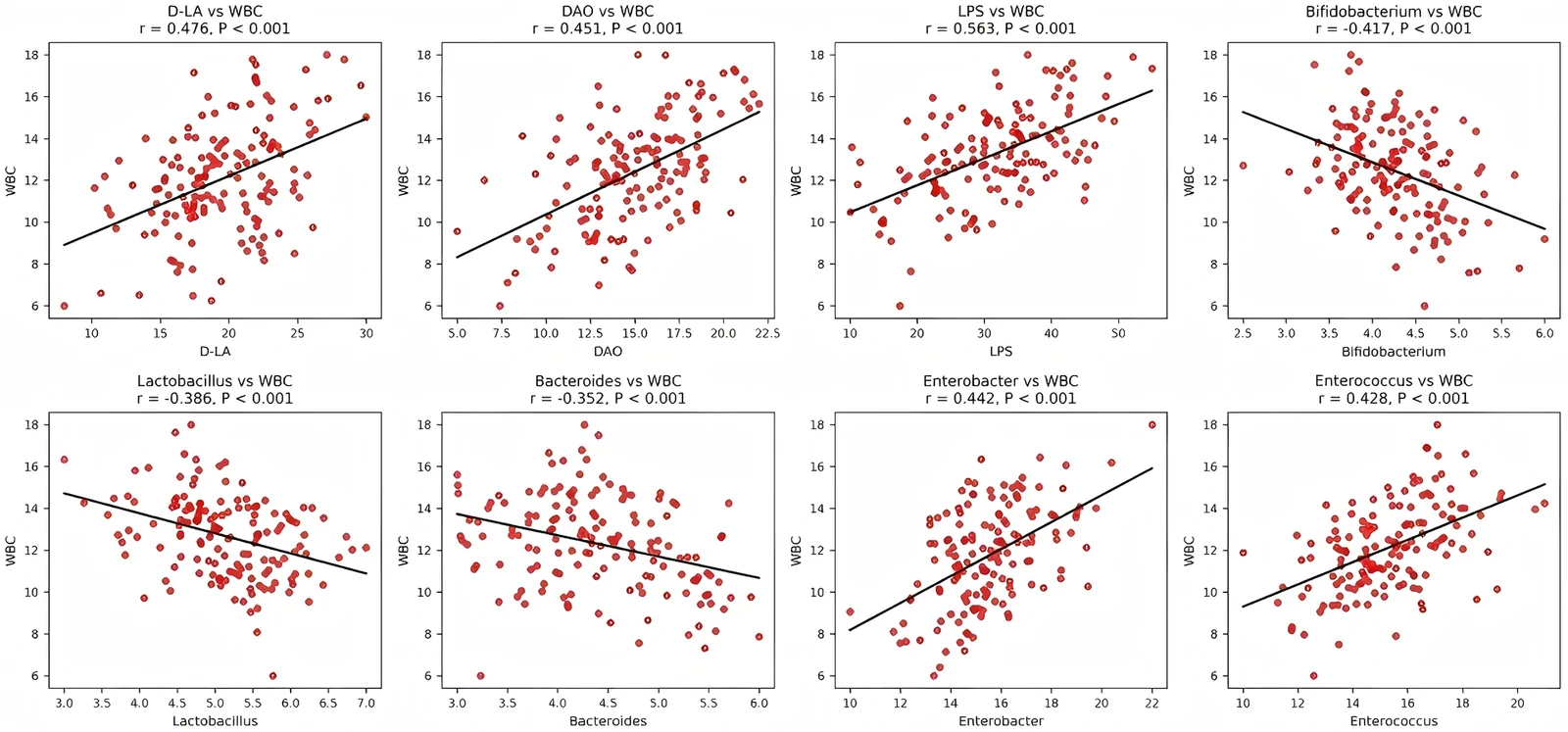
Correlation between gut-related indicators and WBC levels in patients with acute myocardial infarction.

**Figure 7:**
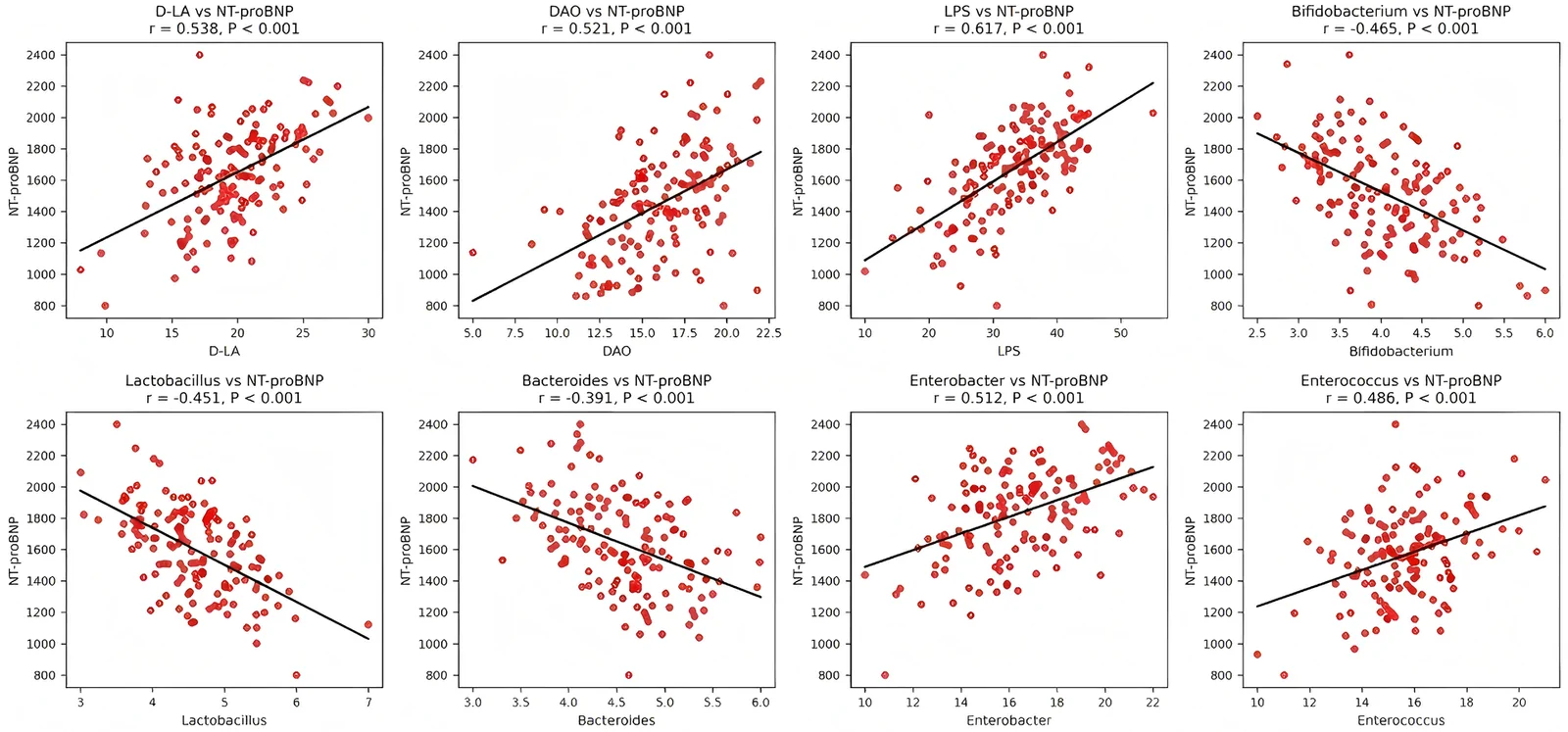
Correlation between gut-related indicators and NT-proBNP levels in patients with acute myocardial infarction.

**Figure 8:**
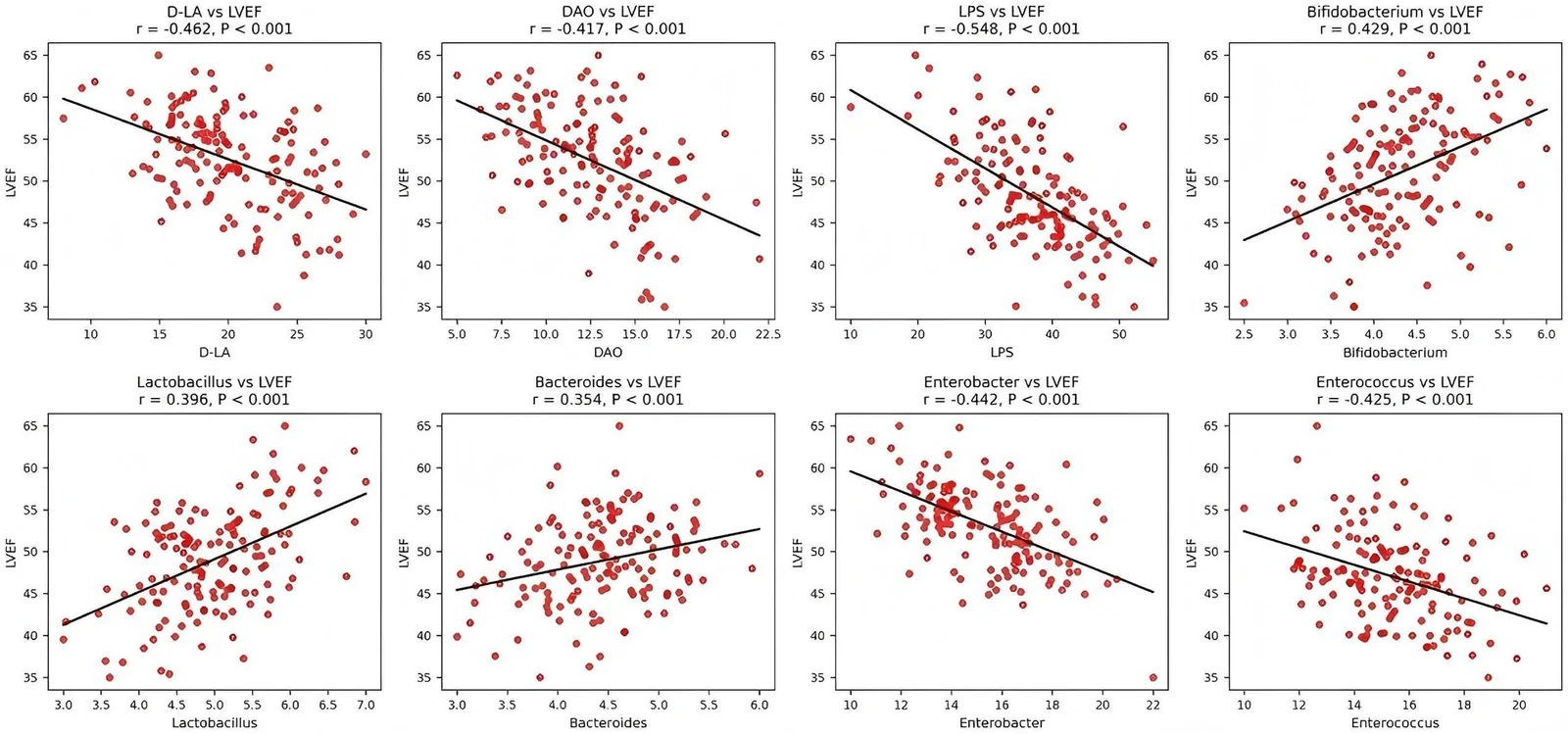
Correlation between gut-related indicators and LVEF in patients with acute myocardial infarction.

Pearson correlation analysis showed biologically consistent correlation patterns between gut-related indicators and inflammatory/cardiac function indicators. D-LA, DAO, LPS, Enterobacter, and Enterococcus were positively correlated with CRP, WBC, and NT-proBNP levels and negatively correlated with LVEF; Bifidobacterium, Lactobacillus, and Bacteroides showed the opposite correlation patterns. The absolute correlation coefficients ranged from 0.352 to 0.617, indicating mostly moderate correlations. Therefore, these results suggest that intestinal barrier dysfunction and intestinal microecological imbalance were associated with enhanced inflammatory response and deterioration of cardiac function, but should not be interpreted as strong predictive effects based on correlation analysis alone (Table 5). To further improve the visualization of these correlation patterns, a heatmap was added to show the direction and relative magnitude of the correlations between gut-related indicators and inflammatory/cardiac function indicators (Figure 4). The original scatter plots were retained to show the distribution of individual data points and the linear trends of each pairwise correlation analysis (Figures 5–8). After Benjamini-Hochberg FDR correction for the 32 correlation tests, all correlations remained statistically significant, with FDR-adjusted q values ＜ 0.001. The detailed nominal *P*-values and FDR-adjusted q values are provided in Supplementary Table S1.

**Table 5:** Pearson correlations between Gut-related indicators and inflammatory and cardiac function indicators.

Indicators	CRP	WBC	NT-proBNP	LVEF
D-LA	0.514	0.476	0.538	−0.462
DAO	0.483	0.451	0.521	−0.417
LPS	0.592	0.563	0.617	−0.548
Bifidobacterium	−0.438	−0.417	−0.465	0.429
Lactobacillus	−0.402	−0.386	−0.451	0.396
Bacteroides	−0.376	−0.352	−0.391	0.354
Enterobacter	0.468	0.442	0.512	−0.442
Enterococcus	0.447	0.428	0.486	−0.425

Values are Pearson correlation coefficients. The Benjamini-Hochberg false discovery rate correction was applied to all 32 correlation tests. All correlations remained statistically significant after correction (all q < 0.001). Detailed nominal *P*-values and FDR-adjusted q values are provided in Supplementary Table S1. The correlations were mostly moderate in magnitude and should not be interpreted as evidence of strong predictive effects.

### 3.6 Exploratory multivariable logistic regression analysis of short-term poor prognosis in AMI patients

The occurrence of MACE within 90 d in AMI patients was used as the dependent variable (no occurrence=0, occurrence=1). During follow-up, 32 patients experienced at least one MACE in total. Candidate variables with statistical significance in univariate analysis, including CRP, WBC, D-LA, DAO, LPS, Bifidobacterium, Lactobacillus, Bacteroides, Enterobacter, Enterococcus, NT-proBNP, and LVEF, were initially included in the multivariable Logistic regression model. Because the number of outcome events was limited relative to the number of candidate predictors, the events-per-variable ratio was low, and this model was considered exploratory and potentially affected by overfitting. The results showed that elevated LPS, elevated DAO, elevated NT-proBNP, decreased LVEF, and increased Enterobacter and Enterococcus were associated with short-term poor prognosis in AMI patients (*P* < 0.05), whereas CRP, WBC, D-LA, and some commensal bacterial indicators were not statistically significant after adjustment (P > 0.05) (Table 6). These findings should be interpreted as exploratory association results rather than definitive independent predictive effects.

**Table 6:** Exploratory multivariable logistic regression analysis of short-term poor prognosis in AMI patients.

Factors	*β*	SE	Wald x^2^	P	OR	95%CI
CRP	0.174	0.102	2.908	0.088	1.190	0.974∼1.454
WBC	0.138	0.094	2.156	0.142	1.148	0.955∼1.380
D-LA	0.207	0.116	3.184	0.074	1.230	0.979∼1.545
DAO	0.371	0.145	6.552	0.010	1.449	1.090∼1.826
LPS	0.428	0.132	10.518	0.001	1.534	1.184∼1.988
Bifidobacterium	−0.216	0.137	2.486	0.115	0.806	0.616∼1.055
Lactobacillus	−0.184	0.128	2.067	0.151	0.832	0.647∼1.070
Bacteroides	−0.153	0.119	1.653	0.198	0.858	0.679∼1.084
Enterobacter	0.318	0.121	6.913	0.008	1.374	1.082∼1.744
Enterococcus	0.341	0.136	6.285	0.012	1.406	1.079∼1.833
NT-proBNP	0.286	0.118	5.874	0.015	1.331	1.056∼1.679
LVEF	−0.402	0.153	6.904	0.009	0.669	0.495∼0.903

A total of 32 patients experienced at least one MACE during the 90-d follow-up period. Because multiple candidate predictors were included in the exploratory Logistic regression model, the events-per-variable ratio was limited. Therefore, the regression results should be interpreted as exploratory association estimates rather than as a stable prognostic prediction model.

## 4 Discussion

This study focused on the core issue of gut microbiota translocation after AMI. The results showed that AMI patients had obvious intestinal barrier dysfunction and structural disturbance of the gut microbiota, and these changes were closely associated with enhanced inflammatory response, deterioration of cardiac function, and short-term poor prognosis, suggesting that intestinal factors may participate in disease progression after AMI. The results of this study showed that, compared with healthy controls, serum D-LA, DAO, and LPS levels were significantly increased in the AMI group. D-LA is mainly derived from gut bacterial metabolism and is normally difficult to enter the circulation in large amounts; when intestinal mucosal permeability increases, its serum level can rise significantly (13). DAO is mainly distributed in small intestinal mucosal epithelial cells and can be released into the blood when the intestinal mucosa is damaged or cellular integrity is disrupted (14).

LPS is an important component of the cell wall of Gram-negative bacteria, and its entry into the bloodstream is often regarded as an important manifestation of intestinal endotoxin translocation (15). Therefore, the simultaneous elevation of these three indicators suggests that AMI patients have developed varying degrees of intestinal mucosal barrier dysfunction and endotoxemia during the acute phase. This finding is generally consistent with previous studies (16, 17) suggesting intestinal hypoperfusion and barrier disruption in critically ill patients with cardiovascular diseases. After AMI, acute myocardial ischemia can lead to decreased cardiac output, sympathetic excitation, and peripheral vasoconstriction, resulting in reduced intestinal blood perfusion. The intestinal mucosa is extremely sensitive to ischemia and hypoxia, and even short-term hypoperfusion can cause loosening of epithelial cell junctions, increased mucosal permeability, and weakening of the local immune barrier (18). On this basis, bacterial metabolites and endotoxins in the intestinal lumen are more likely to enter the circulation, thereby inducing or aggravating systemic inflammatory responses. In addition to abnormalities in intestinal barrier indicators, this study also observed marked changes in the gut microbiota structure of AMI patients, manifested by decreased numbers of beneficial bacteria such as Bifidobacterium, Lactobacillus, and Bacteroides, and significantly increased numbers of opportunistic pathogens such as Enterobacter and Enterococcus. Bifidobacterium and Lactobacillus are representative putatively beneficial bacteria that maintain intestinal microecological stability and can exert protective effects by producing short-chain fatty acids, inhibiting pathogen adhesion, enhancing intestinal epithelial barrier function, and regulating immune responses (19). As one of the major anaerobic commensal genera in the intestine, Bacteroides is involved in polysaccharide metabolism and the maintenance of intestinal immune homeostasis (20). However, Bacteroides should not be interpreted as uniformly beneficial. The genus Bacteroides contains multiple species and strains with heterogeneous biological functions. Some members may participate in intestinal metabolic and immune homeostasis, whereas others may be associated with opportunistic infection or inflammatory conditions under specific host or ecological contexts. Therefore, the decrease in Bacteroides observed in this study should be interpreted as a genus-level change in anaerobic commensal bacteria rather than definitive evidence of the loss of a uniformly beneficial bacterial population. In contrast, Enterobacter and Enterococcus are prone to overgrowth when microbiota imbalance, altered intestinal hypoxic environment, or host immune dysfunction occurs, and they may aggravate inflammatory responses by releasing endotoxins, cell wall components, and pro-inflammatory metabolites (21). Previous reviews and mechanistic studies have suggested that gut microbiota dysbiosis and intestinal barrier dysfunction are closely involved in coronary artery disease, heart failure, and related cardiovascular conditions (22, 23). More importantly, previous microbiome sequencing studies have provided direct evidence supporting gut microbiota alterations in atherosclerotic cardiovascular disease. Karlsson et al. used shotgun metagenomic sequencing and reported that patients with symptomatic atherosclerosis had an altered gut metagenome, characterized by enrichment of Collinsella and relative enrichment of Roseburia and Eubacterium in healthy controls (24). In a larger metagenome-wide association study, Jie et al. found that patients with atherosclerotic cardiovascular disease showed increased abundance of Enterobacteriaceae and Streptococcus spp., together with relative depletion of several commensal bacteria and butyrate-producing taxa (25). These sequencing-based findings generally support the dysbiotic pattern observed in the present study, namely a shift toward increased opportunistic/pathobiont bacteria and reduced putatively beneficial or commensal bacteria in patients with cardiovascular disease. However, unlike these sequencing studies, the present study used culture-based detection of selected representative bacteria. Therefore, our findings should be interpreted as clinical culture-based evidence consistent with previous sequencing results rather than as a comprehensive microbiome profiling study.

Because D-LA, DAO, and LPS were used as part of the criteria for defining gut microbiota translocation, their higher levels in the translocation group were expected by design and should be interpreted only as confirmation of the grouping criteria rather than as independent subgroup findings. Therefore, we refocused the subgroup interpretation on gut microbiota indicators and downstream clinical outcomes.

**Figure 9:**
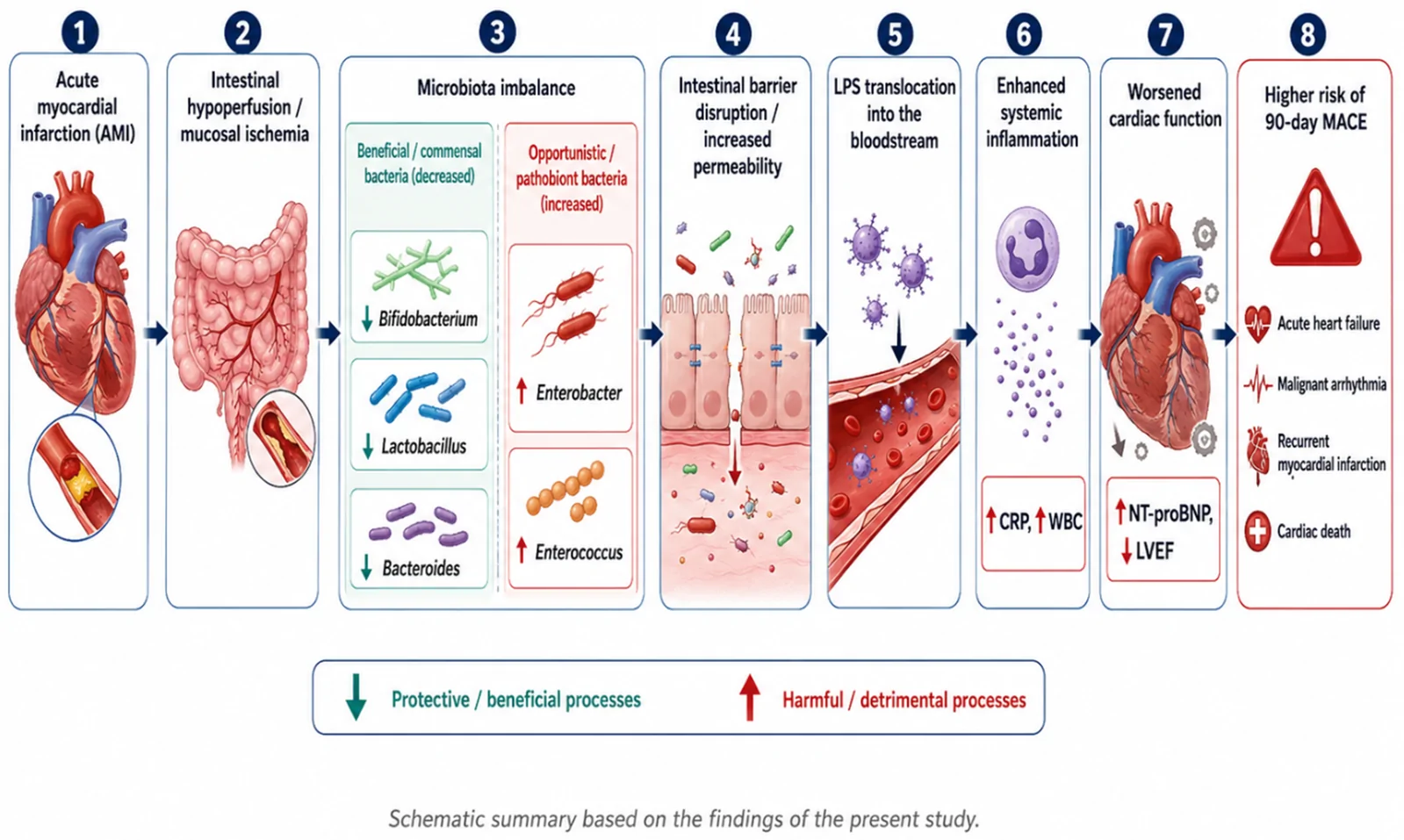
Schematic representation of the proposed changes in beneficial gut bacteria and gut microbiota translocation after acute myocardial infarction. Acute myocardial infarction may lead to intestinal hypoperfusion and mucosal ischemia, which are associated with reductions in putatively beneficial or commensal bacteria (Bifidobacterium, Lactobacillus, and Bacteroides) and increases in opportunistic/pathobiont bacteria (Enterobacter and Enterococcus). These changes may contribute to intestinal barrier disruption, increased permeability, endotoxin translocation (LPS) into the circulation, enhanced systemic inflammation, and subsequent deterioration of cardiac function and short-term prognosis.

Compared with the non-translocation group, the translocation group showed further decreases in Bifidobacterium, Lactobacillus, and Bacteroides and further increases in Enterobacter and Enterococcus, suggesting that microbiota translocation in AMI patients may be accompanied by more pronounced intestinal microecological imbalance. In other words, gut microbiota translocation in AMI patients may involve a continuous process of “reduction of putatively beneficial or commensal bacteria— proliferation of opportunistic/pathobiont bacteria — intestinal mucosal barrier disruption — endotoxin entry into the bloodstream.” A brief schematic representation of this proposed process is shown in Figure 9. Compared with the non-translocation group, CRP and WBC levels were significantly increased in the translocation group, which further supports the close association between microbiota translocation and enhanced systemic inflammatory response. After entering the circulation, LPS can activate Toll-like receptor-related signaling pathways and promote monocytes/macrophages and endothelial cells to release inflammatory factors, thereby amplifying systemic inflammatory responses (26). AMI itself is accompanied by sterile inflammation induced by myocardial necrosis, while intestinal endotoxin translocation may further amplify the inflammatory cascade, shifting the body from a state of local myocardial injury to broader systemic inflammatory activation (27). This study also found that NT-proBNP levels were significantly higher and LVEF was significantly lower in the translocation group than in the non-translocation group, suggesting that patients with gut microbiota translocation had more severe cardiac function impairment. NT-proBNP is an important indicator reflecting ventricular wall tension and cardiac functional load, and its elevation often indicates an increased risk of cardiac insufficiency (28). LVEF is an important parameter for evaluating left ventricular systolic function (29). A bidirectional relationship may exist between gut microbiota translocation and deterioration of cardiac function. On the one hand, decreased cardiac function after AMI can lead to intestinal hypoperfusion and venous congestion, further aggravating intestinal mucosal edema, hypoxia, and barrier disruption, thereby promoting microbiota translocation. On the other hand, microbiota translocation and endotoxemia can aggravate myocardial injury and promote ventricular remodeling and cardiac function decline through inflammatory responses, oxidative stress, and endothelial dysfunction.

This mutually reinforcing relationship may form a vicious cycle of “cardiac function impairment — intestinal barrier disruption — microbiota translocation — enhanced inflammatory response—further deterioration of cardiac function” (30). Previous studies (31) on intestinal congestion and intestinal-derived inflammation in patients with heart failure have also suggested that intestinal barrier dysfunction may be an important contributor to the progression of cardiac insufficiency. The present study advances this mechanism to the acute phase of AMI, suggesting that intestinal factors may already play a role in early cardiac functional outcomes after AMI. Correlation analysis further verified the internal links among the above results. D-LA, DAO, LPS, Enterobacter, and Enterococcus were positively correlated with CRP, WBC, and NT-proBNP and negatively correlated with LVEF; Bifidobacterium, Lactobacillus, and Bacteroides showed the opposite trends. This indicates that the more obvious the intestinal barrier injury and the greater the abundance of opportunistic pathogens, the higher the inflammatory level and the more severe the cardiac function impairment; whereas higher levels of putatively beneficial or commensal bacteria at the genus level are associated with relatively milder inflammatory responses and cardiac dysfunction. Notably, LPS showed relatively stronger correlations with inflammatory and cardiac function indicators, suggesting that endotoxemia may be an important intermediate link connecting gut microbiota imbalance with adverse cardiac functional outcomes after AMI. D-LA and DAO mainly reflect the status of barrier function, whereas LPS further reflects the systemic effects after intestinal-derived harmful components enter the circulation. Therefore, when evaluating microbiota translocation in AMI patients, focusing solely on changes in microbiota abundance may be insufficient; combined detection of intestinal barrier injury indicators and endotoxin levels may better reflect its clinical significance.

Follow-up results showed that the incidence of MACE within 90 d was significantly higher in the translocation group than in the non-translocation group, among which the increases in acute heart failure and malignant arrhythmia were particularly prominent. This finding suggests that gut microbiota translocation is not only associated with inflammatory status and cardiac function at admission but is also associated with short-term prognosis. The early stage after AMI is a critical period for cardiac functional changes and ventricular remodeling, during which persistent inflammatory responses, endothelial dysfunction, neuroendocrine activation, and decreased myocardial electrical stability may all increase the risk of adverse events (32). Endotoxemia and inflammatory amplification induced by microbiota translocation may promote cardiomyocyte injury, interstitial fibrosis, and electrophysiological abnormalities, thereby increasing the risk of heart failure and malignant arrhythmias. Compared with previous studies mainly focusing on prognostic indicators such as NT-proBNP, LVEF, and infarct size, the present study provides new evidence for short-term prognostic evaluation of AMI from the perspectives of intestinal barrier dysfunction and microbiota translocation, suggesting that gut-related indicators may serve as auxiliary risk stratification tools beyond traditional cardiac function assessment. Exploratory multivariable Logistic regression analysis showed that after incorporating inflammatory indicators, intestinal barrier indicators, microbiota indicators, and cardiac function indicators, elevated LPS, elevated DAO, elevated NT-proBNP, decreased LVEF, and increased Enterobacter and Enterococcus were associated with short-term poor prognosis in AMI patients. However, because only 32 MACE events occurred during follow-up and multiple candidate predictors were included, the events-per-variable ratio was low, and the regression coefficients and confidence intervals may be unstable. Therefore, these findings should be interpreted as exploratory associations rather than definitive independent predictors. These exploratory findings suggest that the associations between gut microbiota translocation-related indicators and prognosis may not be fully captured by traditional cardiac function indicators alone. Elevated DAO suggests more severe intestinal mucosal injury, elevated LPS indicates increased risk of endotoxemia and microbiota translocation, and increased Enterobacter and Enterococcus reflect the predominance of opportunistic pathogens.

Together, these factors point to the potential role of the intestinal barrier –microbiota—inflammation axis in the prognostic evolution after AMI. Although inflammatory indicators such as CRP and WBC were closely associated with intestinal indicators in univariate or correlation analyses, they were not statistically significant after adjustment in the exploratory multivariable model, possibly due to overlapping effects with LPS, DAO, and cardiac function indicators. These findings suggest that intestinal barrier injury and endotoxin translocation may be clinically relevant to disease progression, but their independent predictive value requires validation in larger prospective cohorts.

From a clinical perspective, the results of this study suggest that, in addition to routine assessment of myocardial injury, cardiac function, and inflammatory status after admission, appropriate attention to intestinal barrier function and microbiota status may help more comprehensively identify high-risk AMI patients. Detection of D-LA, DAO, and LPS is relatively simple, and combined with representative gut microbiota culture results, may provide useful evidence for evaluating gut microbiota translocation and microecological imbalance. For patients with obvious intestinal barrier dysfunction, elevated LPS levels, and increased opportunistic pathogens, greater attention may need to be paid clinically to inflammatory control, improvement of circulatory perfusion, nutritional support, and maintenance of intestinal function. If future prospective studies further confirm the effectiveness of gut microecological interventions, probiotics, prebiotics, dietary regulation, and intestinal barrier-protective strategies may become supplementary approaches in the comprehensive management of AMI. However, it still cannot be simply assumed that improving the microbiota can directly improve AMI prognosis. The underlying mechanisms, timing of intervention, target population, and safety still require further investigation and validation.

In addition, this study has several limitations. (1) This was a single-center retrospective cohort study conducted in Tianjin. Therefore, the findings may be influenced by selection bias, information bias, center-specific clinical practice patterns, and local laboratory workflows. In addition, the AMI cohort included a relatively high proportion of male patients, which may limit the applicability of the findings to female patients with AMI. Because dietary habits, gut microbiota composition, clinical testing indications, and treatment pathways may differ across regions, hospital types, and patient populations, the present results should not be directly generalized to all AMI populations. (2) The sample size was relatively limited, and no formal *a priori* sample size or power calculation was performed. The final sample size was determined by the number of eligible subjects with complete clinical, laboratory, gut microbiota, and follow-up data during the predefined study period. Similarly the subgroup sizes of the translocation and non-translocation groups were determined according to the predefined translocation criteria rather than by prospective sample size planning. In addition, only 32 patients experienced at least one MACE during the 90-d follow-up, whereas multiple candidate predictors were included in the Logistic regression analysis. This resulted in a low events-per-variable ratio and may have increased the risk of model overfitting, unstable regression coefficients, and overly optimistic confidence intervals. Therefore, the regression results should be regarded as exploratory rather than confirmatory. (3) This study used culture-based methods to detect five representative gut microbiota. Although this approach is simple and clinically practical, it provides only limited genus-level information and cannot comprehensively reflect gut microbiota diversity, microbial function, or metabolite-related changes. In particular, culture-based detection could not distinguish different species or strains within Bacteroides, whose members may have distinct biological roles under different host and ecological conditions. Therefore, the terms “ putatively beneficial, “ “ commensal, “ and “ opportunistic/pathobiont “ should be interpreted only as descriptive genus-level categories rather than definitive functional or pathogenic classifications. In addition, because this retrospective study was based on existing routine clinical laboratory records, PCR-based detection of genes involved in beneficial metabolite production or strain-specific molecular markers was not available. Consequently, the functional potential and strain-level identity of the cultured bacteria could not be molecularly verified, and organisms with potentially different biological effects may have been grouped within the same genus. This limitation reduces the biological specificity of the microbiota findings and prevents the observed clinical associations from being attributed to particular microbial genes, functional pathways, or strains. Therefore, although it does not negate the observed culture-based associations with intestinal barrier dysfunction, inflammatory response, cardiac function, and short-term outcomes, the present results and conclusions should be interpreted as genus-level clinical associations rather than evidence of strain-specific microbial functions or gene-mediated mechanisms. (4) The follow-up period was limited to 90 d, and this study mainly focused on short-term adverse cardiovascular events. In addition, serial symptom scores and symptom-duration data were not consistently available in the retrospective medical records; therefore, the persistence or temporal continuity of cardiac symptoms could not be evaluated separately. Therefore, the effects of gut microbiota translocation on long-term ventricular remodeling, chronic heart failure, recurrent cardiovascular events, and mortality risk remain unclear. Future studies with longer follow-up are needed to determine whether gut-related indicators have sustained prognostic value after AMI. (5) Although multiple clinical indicators were included in the analysis, residual confounding could not be completely excluded. Factors such as dietary structure, nutritional status during hospitalization, intestinal motility, medication use, occult infection, and other unmeasured clinical variables may have influenced intestinal barrier function, gut microbiota status, inflammatory response, and prognosis. Moreover, microbiota-mediated drug metabolism may interact with medication exposure and should be considered in future studies (33). Therefore, the results of this study should be interpreted as evidence of clinical association rather than direct causal conclusions. Future multicenter prospective studies with larger sample sizes, more diverse populations, longer follow-up, prespecified reduced models or penalized regression methods, and integrated analyses and integrated analyses combining culture-based isolation, PCR-based assays, 16S rRNA sequencing, metagenomic sequencing, and metabolomics are needed to further validate these findings.

## 5 Conclusion

AMI patients in the acute phase exhibit obvious intestinal barrier dysfunction and gut microbiota translocation, mainly manifested by elevated levels of D-LA, DAO, and LPS, decreased putatively beneficial or commensal bacteria and increased opportunistic/pathobiont bacteria. Gut microbiota translocation is closely associated with enhanced inflammatory response, deterioration of cardiac function, and the occurrence of MACE within 90 d. Among these indicators, LPS, DAO, Enterobacter, and Enterococcus showed potential associations with short-term poor prognosis in exploratory regression analysis, but their independent predictive value requires further validation in larger cohorts with more outcome events. These genus-level, culture-based findings support the possibility that the “gut-heart axis “participates in disease progression and prognostic evolution after AMI, and provide preliminary clinical evidence for risk stratification and future gut microecology-related research in AMI patients.

## Data Availability

The original contributions presented in the study are included in the article/Supplementary Material, further inquiries can be directed to the corresponding author.

## References

[1] Saito Y Oyama K Tsujita K Yasuda S Kobayashi Y. Treatment strategies of acute myocardial infarction: updates on revascularization, pharmacological therapy, and beyond. J Cardiol. (2023) 81(2):168–78. 10.1016/j.jjcc.2022.07.003 35882613

[2] Sawano S Sakakura K Taniguchi Y Yamamoto K Tsukui T Seguchi M. Outcomes of patients with acute myocardial infarction who recovered from severe in-hospital complications. Am J Cardiol. (2020) 135:24–31. 10.1016/j.amjcard.2020.08.031 32871110

[3] Ramachandra CJA Hernandez-Resendiz S Crespo-Avilan GE Lin Y-H Hausenloy DJ. Mitochondria in acute myocardial infarction and cardioprotection. EBioMedicine. (2020) 57:102884. 10.1016/j.ebiom.2020.102884 32653860 PMC7355051

[4] Zhu L Liu Y Wang K Wang N. Regulated cell death in acute myocardial infarction: molecular mechanisms and therapeutic implications. Ageing Res Rev. (2025) 104:102629. 10.1016/j.arr.2024.102629 39644925

[5] Gao J Zhang M Zhang G Zhang D Zhou M Zhao L. Advances in nutritional interventions for coronary heart disease patients from the perspective of the gut-heart axis. Front Nutr. (2025) 12:1676619. 10.3389/fnut.2025.1676619 41323992 PMC12661857

[6] Gomaa EZ. Human gut microbiota/microbiome in health and diseases: a review. Antonie Van Leeuwenhoek. (2020) 113(12):2019–40. 10.1007/s10482-020-01474-7 33136284

[7] Chen Y Zhou J Wang L. Role and mechanism of gut Microbiota in human disease. Front Cell Infect Microbiol. (2021) 11:625913. 10.3389/fcimb.2021.625913 33816335 PMC8010197

[8] Robles-Vera I Jarit-Cabanillas A Brandi P Martínez-López M Martínez-Cano S Rodrigo-Tapias M. Microbiota translocation following intestinal barrier disruption promotes mincle-mediated training of myeloid progenitors in the bone marrow. Immunity. (2025) 58(2):381–396.e9. 10.1016/j.immuni.2024.12.012 39848243 PMC11832192

[9] Tilg H Zmora N Adolph TE Elinav E. The intestinal microbiota fuelling metabolic inflammation. Nat Rev Immunol. (2020) 20(1):40–54. 10.1038/s41577-019-0198-4 31388093

[10] Habes QLM Kant N Beunders R Van Groenendael R Gerretsen J Kox M. Relationships between systemic inflammation, intestinal damage and postoperative organ dysfunction in adults undergoing low-risk cardiac surgery. Heart Lung Circ. (2023) 32(3):395–404. 10.1016/j.hlc.2022.12.006 36621395

[11] Byrne RA Rossello X Coughlan JJ Barbato E Berry C Chieffo A. 2023 ESC guidelines for the management of acute coronary syndromes. Eur Heart J. (2023) 44(38):3720–826. 10.1093/eurheartj/ehad191 37622654

[12] Takasaki A Kurita T Hirabayashi Y Matsuo H Tanoue A Masuda J. Prognosis of acute myocardial infarction in patients on hemodialysis stratified by killip classification in the modern interventional era (focus on the prognosis of Killip class 1). Heart Vessels. (2022) 37(2):208–18. 10.1007/s00380-021-01919-7 34347137

[13] Pohanka M. D-Lactic acid as a metabolite: toxicology, diagnosis, and detection. Biomed Res Int. (2020):9. 10.1155/2020/3419034 PMC7320276 32685468

[14] Rentzos G Weisheit A Ekerljung L Van Odijk J. Measurement of diamine oxidase (DAO) during low-histamine or ordinary diet in patients with histamine intolerance. Eur J Clin Nutr. (2024) 78(8):726–31. 10.1038/s41430-024-01448-2 38769188 PMC11300302

[15] Meng M Huo R Wang Y Ma N Shi X Shen X. Lentinan inhibits oxidative stress and alleviates LPS-induced inflammation and apoptosis of BMECs by activating the Nrf2 signaling pathway. Int J Biol Macromol. (2022) 222(PtB):2375–91. 10.1016/j.ijbiomac.2022.10.024 36243161

[16] Kondapalli N Katari V Dalal KK Paruchuri S Thodeti CK. Microbiota in gut-heart axis: metabolites and mechanisms in cardiovascular disease. Compr Physiol. (2025) 15(3):e70024. 10.1002/cph4.70024 40542540 PMC12181760

[17] Li D Chen F. Efects of gut Microbiota on hypertension and the cardiovascular system. Nutrients. (2023) 15(21):4633. 10.3390/nu15214633 37960287 PMC10650547

[18] Archontakis-Barakakis P Mavridis T Chlorogiannis D-D Barakakis G Laou E Sessler DI. Intestinal oxygen utilisation and cellular adaptation during intestinal ischaemia-reperfusion injury. Clin Transl Med. (2025) 15(1):e70136. 10.1002/ctm2.70136 39724463 PMC11670310

[19] Xiao Y Zhai Q Zhang H Chen W Hill C. Gut colonization mechanisms of Lactobacillus and Bifidobacterium: an argument for personalized designs. Annu Rev Food Sci Technol. (2021) 12:213–33. 10.1146/annurev-food-061120-014739 33317320

[20] Kang Z Jiang S Fang J-Y Chen H. Intestinal dysbiosis and colorectal cancer. Chin Med J (Engl). (2025) 138(11):1266–87. 10.1097/CM9.0000000000003617 40387510 PMC12150945

[21] Quaglio AEV Grillo TG Oliveira ECSD Stasi LCD Sassaki LY. Gut microbiota, inflammatory bowel disease and colorectal cancer. World J Gastroenterol. (2022) 28(30):4053–60. 10.3748/wjg.v28.i30.4053 36157114 PMC9403435

[22] Trøseid M Andersen GØ Broch K Hov JR. The gut microbiome in coronary artery disease and heart failure: current knowledge and future directions. EBioMedicine. (2020) 52:102649. 10.1016/j.ebiom.2020.102649 PMC7016372 32062353

[23] Lewis CV Taylor WR. Intestinal barrier dysfunction as a therapeutic target for cardiovascular disease. Am J Physiol Heart Circ Physiol. (2020) 319(6):H1227–33. 10.1152/ajpheart.00612.2020 PMC7792706 32986965

[24] Karlsson FH Fåk F Nookaew I Tremaroli V Fagerberg B Petranovic D. Symptomatic atherosclerosis is associated with an altered gut metagenome. Nat Commun. (2012) 3:1245. 10.1038/ncomms2266 23212374 PMC3538954

[25] Jie Z Xia H Zhong S-L Feng Q Li S Liang S. The gut microbiome in atherosclerotic cardiovascular disease. Nat Commun. (2017) 8:845. 10.1038/s41467-017-00900-1 29018189 PMC5635030

[26] Li Q Tan Y Chen S Xiao X Zhang M Wu Q. Irisin alleviates LPS-induced liver injury and inflammation through inhibition of NLRP3 inflammasome and NF-*κ*B signaling. J Recept Signal Transduct Res. (2021) 41(3):294–303. 10.1080/10799893.2020.1808675 32814473

[27] Zhao J Zhang Q Cheng W Dai Q Wei Z Guo M. Heart-gut microbiota communication determines the severity of cardiac injury after myocardial ischaemia/reperfusion. Cardiovasc Res. (2023) 119(6):1390–402. 10.1093/cvr/cvad023 PMC10262181 36715640

[28] Bayes-Genis A Docherty KF Petrie MC Januzzi JL Mueller C Anderson L. Practical algorithms for early diagnosis of heart failure and heart stress using NT-proBNP: a clinical consensus statement from the heart failure association of the ESC. Eur J Heart Fail. (2023) 25(11):1891–8. 10.1002/ejhf.3036 37712339

[29] Baumhove L Tromp J Figarska S Van Essen BJ Anker SD Dickstein K. Heart failure with normal LVEF in BIOSTAT-CHF. Int J Cardiol. (2022) 364:85–90. 10.1016/j.ijcard.2022.05.054 35649488

[30] Rivera K Gonzalez L Bravo L Manjarres L Andia ME. The gut-heart axis: molecular perspectives and implications for myocardial infarction. Int J Mol Sci. (2024) 25(22):12465. 10.3390/ijms252212465 39596530 PMC11595032

[31] Chen AT Zhang J Zhang Y. Gut microbiota in heart failure and related interventions. Imeta. (2023) 2(3):e125. 10.1002/imt2.125 38867928 PMC10989798

[32] Patti G Cumitini L Bosco M Marengo A D'Amario D Mennuni M. Impact of a personalized, strike early and strong lipid-lowering approach on low-density lipoprotein-cholesterol levels and cardiovascular outcome in patients with acute myocardial infarction. Eur Heart J Cardiovasc Pharmacother. (2025) 11(2):143–54. 10.1093/ehjcvp/pvaf004 39855642 PMC11905752

[33] Kapoor S Sahoo AK Yadav AK. Role of gut microbiota in the drug metabolism: a review. Innov Discov. (2026) 3(1):2. 10.53964/id.2026002

